# In Vitro Antiviral Evaluations of Coldmix^®^: An Essential Oil Blend against SARS-CoV-2

**DOI:** 10.3390/cimb45010045

**Published:** 2023-01-11

**Authors:** Kemal Hüsnü Can Başer, Ayşe Esra Karadağ, Sevde Nur Biltekin, Murat Ertürk, Fatih Demirci

**Affiliations:** 1 Department of Pharmacognosy, Faculty of Pharmacy, Near East University, N. Cyprus, Mersin 10, 99138 Nicosia, Türkiye; 2 Badebio Biotechnology Ltd., ATAP, Anadolu University, Tepebaşı, 26470 Eskişehir, Türkiye; 3 Department of Pharmacognosy, School of Pharmacy, Istanbul Medipol University, 34810 Istanbul, Türkiye; 4 Department of Pharmacognosy, Graduate School of Health Sciences, Anadolu University, 26470 Eskişehir, Türkiye; 5 Department of Pharmaceutical Microbiology, School of Pharmacy, Istanbul Medipol University, 34810 Istanbul, Türkiye; 6 Department of Molecular Biology and Genetics, Faculty of Science, Istanbul University, 34134 Istanbul, Türkiye; 7 Department of Microbiology, Medical School of Yüksek İhtisas University, 06520 Ankara, Türkiye; 8 Department of Pharmacognosy, Faculty of Pharmacy, Anadolu University, 26470 Eskişehir, Türkiye; 9 Faculty of Pharmacy, Eastern Mediterranean University, N. Cyprus, Mersin 10, 99628 Famagusta, Türkiye

**Keywords:** *Eucalyptus*, *Abies*, essential oils, ACE2, coronavirus, antiviral

## Abstract

Coldmix^®^ is a commercially available *Eucalyptus aetheroleum* and, *Abies aetheroleum* blend for medicinal applications. In this present study, the in vitro antiviral potential of Coldmix^®^, and its major constituents 1,8-cineole and α-pinene were evaluated by using the in vitro ACE2 enzyme inhibition assay as well as the direct contact test against SARS-CoV-2. The observed ACE2 enzyme inhibitory activity of Coldmix^®^, 1,8-cineole, and α-pinene were 72%, 88%, and 80%, respectively; whereas in the direct contact test in the vapor phase, the destruction of the virus was 79.9% within 5 min and 93.2% in the 30th min, respectively. In a similar Coldmix^®^ vapor phase setup using the in vitro cytotoxicity cell assay, E6 VERO healthy cells were experimentally not affected by toxicity. According to the promising initial antiviral results of Coldmix^®^ and the individually tested constituents, detailed further in vivo evaluation using different virus classes is suggested.

## 1. Introduction

Traditionally, *Eucalyptus* and *Abies* essential oils are used specifically to treat respiratory complaints and disorders. It is well documented that *Eucalyptus* essential oils and preparations are also used for the treatment of pharyngitis, bronchitis, and sinusitis [1,2,3,4,5]. In previous studies, results showing the antimicrobial and antiviral effects of *Eucalyptus* essential oil were reported [6,7]. It is well documented that *Eucalyptus* essential oils are prominent with their antimicrobial activities, especially against upper respiratory tract pathogens and infections [8,9,10,11,12]. The *Eucalyptus* oil was previously demonstrated to be effective against influenza and herpes viruses [13,14]. In addition, the antimicrobial effect of *Eucalyptus* essential oil combinations was found to be effective, in particular against upper respiratory tract diseases [15]. Both the *Eucalyptus* oil and its active component 1,8-cineole was observed for its muscle relaxant effect by reducing the smooth muscle contractions in respiratory disorders [16,17,18]. It was also reported that 1,8-cineole was effective against influenza A (H1N1), and inactivated the free influenza A virus by disruption of the envelope structures in vitro [15]. A previous study demonstrated the efficacy of 1,8-cineole in numerous clinical studies conducted in patients with acute and chronic respiratory conditions, including rhinosinusitis, bronchitis, COPD and asthma [18,19].

The inhalation of *Abies* species preparations showed effective results, especially in microbial and viral infections of the respiratory tract [20,21]. *Abies* species were inhibitory against upper respiratory pathogenic bacteria, and this effect was observed to increase even more in *Abies* species rich in α-pinene content [22,23,24,25,26]. The monoterpenes α-pinene as well as 1,8-cineole were previously reported for their in vitro antiviral activity [27]. 1,8-Cineole was recently demonstrated in vitro as an ACE2 inhibitor [28]. ACE2 was one of the receptors causing SARS-CoV-2 [29]. Previous studies reported also that ACE2 is one of the essential receptors for various coronavirus types for cell entry [30,31].

Recent in silico studies showed that essential oils may be effective against COVID-19 by various volatile components [31,32]. The potential effects of *Eucalyptus* oil and its major constituent 1,8-cineole were recently evaluated against SARS-CoV-2 using in vitro tests and molecular docking techniques [33,34], which suggested the detailed experimentation of volatile compounds and essential oils against coronaviruses.

Effective measures and approaches, such as the application of small-molecule inhibitors and vaccines are investigated to reduce SARS-CoV-2 transmission [35,36]; however, effective and promising drugs still do not exist [37]. As an indispensable resource, traditional medicines [38] and bioactive natural products [39] demonstrated a potential value in countering SARS-CoV-2 infection. In this present study, the in vitro antiviral activity and ACE2 enzyme inhibitory potential of the commercial medical device Coldmix^®^, and its major constituents 1,8-cineole and α-pinene were studied, to the best of our knowledge for the first time.

## 2. Materials and Methods

### 2.1. Materials

If not stated otherwise, all chemicals such as 1,8-cineole, α-pinene, and DMSO were acquired from Sigma/Aldrich at analytical or pharmaceutical grade; the enzyme kits, standard materials and Coldmix^®^ were provided by ENAFARMA Ltd., Istanbul, Turkey.

GC-FID and GC/MS analysis details of Coldmix^®^: α-pinene: 13.7 ± 0.9%, and 1,8-cineole: 30.7 ± 0.8%.

### 2.2. ACE2 Enzyme Inhibitory Activity

**Figure 1:**
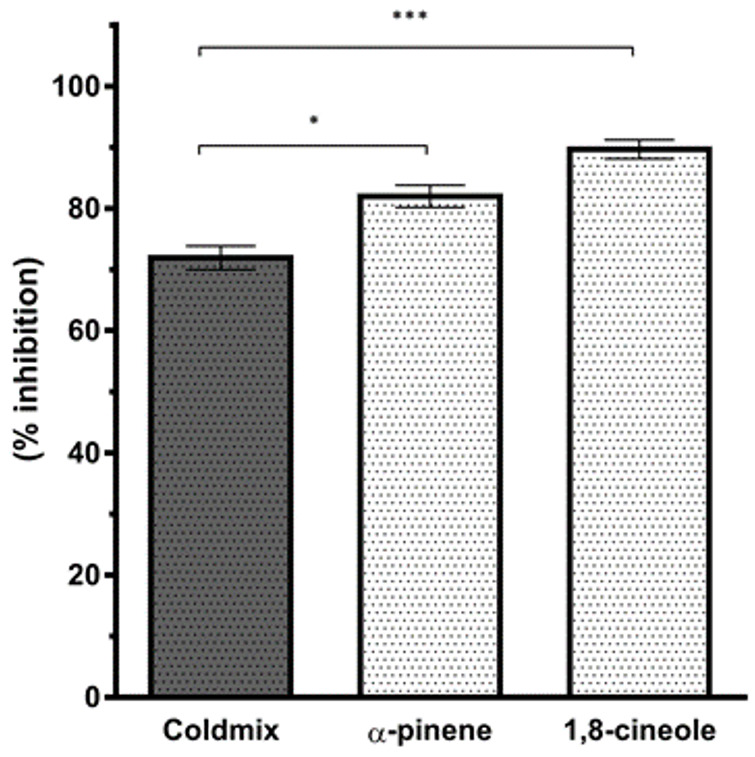
The in vitro ACE2 inhibition graph of Coldmix^®^ and major components, presenting the mean ± SD values (*n* = 6). * *p* < 0.05 *** *p* < 0.001 determined by one-way ANOVA using Dunnet’s multiple comparison test.

The test substances were initially dissolved in DMSO < 1% (*v*/*v*). The enzyme inhibition experiment was carried out in accordance with the manufacturer’s instructions for the kit “Angiotensin II Converting Enzyme (ACE2) Inhibitor Screening Kit (BioVision, K310 (Waltham, MA, USA))” and the enzyme inhibition of the substances was measured with Ex/Em = 320/420 nm wavelength in a multimode microplate reader (SpectraMax i3 (Sunnyvale, CA, USA)) at fluorescence mode. % inhibition values were calculated for all samples resulting from duplicate data as previously reported [28,40]. Results were illustrated in Figure 1.

### 2.3. In Vitro Antiviral Activity

The antiviral effectiveness of Coldmix^®^ was tested against SARS-CoV-2 virus (Clinical isolate, GenBank No: MT955161.1) using the Biosafety Level 3 (BSL3) facility at the Antimikrop Ar-Ge Biyosidal Merkezi (Ankara, Turkey), a research center with ISO/IEC 17025 accreditation, which is also licensed by the Turkish Ministry of Health as a competent laboratory for biocidal testing. Virus stock culture was prepared using the Vero E6 cell line, propagated using DMEM-10 (Dulbecco’s Minimum Essential Medium) supplemented with 10% FBS (fetal bovine serum) and pen/strep/fungizone.

The virus suspension was spotted onto the 2 cm^2^ area on the stainless steel disc, as described in TS EN 16777: 2019 standard, and left to dry in the biosafety cabinet [TS EN 16777:2018: Chemical Disinfectants and Antiseptics—Quantitative Non-Porous Surface Test without Mechanical Action for the Evaluation of Virucidal Activity of Chemical Disinfectants Used in the Medical Area—Test Method and Requirements (Phase 2/Step 2) European Committee for Standardization; Brussels, Belgium: 2019 2019)]. After the discs were dried, 4 control discs were left in the biosafety cabinet, while 6 test discs were placed in a 15 L plastic container with a lid. A piece of cloth fabric onto which 5–6 drops of Coldmix^®^ applied was also placed into one corner of the box, making sure that the virus containing discs were about 20 cm away from the cloth. After tightly closing the box with its lid, it was placed into an incubator for 5 and 30 min at 30 ± 1 °C. The control disks were kept in a sealed petri dish at the lower shelves of the incubator. After the exposure times, disks were individually processed as described in the EN 16777: 2019 standard, by performing an extraction procedure which essentially involves vortexing the discs for 30 s in 0.9 mL complete DMEM. The harvest of each disc was then serially diluted in 10-folds using complete DMEM, and transferred into a 96-well tissue culture plate containing Vero E6 cell suspension at 20,000 cell/well. The plates were incubated at 37 ± 10 °C, 5% CO_2_ environment for 4 days [41,42,43]. The 50% tissue culture infective dose (TCID_50_) of the tests and controls were calculated using the Spearman-Kärber method, as described by EN 16777: 2019 standard. The titers were expressed as log10 TCID_50_.

### 2.4. Cytotoxicity

To measure the extent of the cellular toxicity, the cells were directly exposed to the vapor of Coldmix^®^ in a closed chamber. For this, E6 VERO cell suspension was seeded into 96-well plates at 20,000 cells/well and incubated in a 37 °C 5% CO_2_ incubator overnight. The next day, a water wetted tissue paper was placed in a 15 L plastic container with a lid, and kept in an incubator at 37 °C and 90% humidity. 10 × 10 cm fabric was placed inside the container by folding it in half in a corner. 6–8 drops of Coldmix^®^ were dropped on the fabric, and the lid of the box was closed and placed into 37 °C incubator for vapor conditioning inside the container. After five minutes, the contents of the cell plates was replaced with 0.025 mL/well of DMEM containing 2% FBS to prevent drying of cell layer during the incubation period. Subsequently, half of the plates were covered with an adhesive plate cover to avoid exposure to Coldmix^®^ vapor. The closed part was used as a control. The plates were then placed into the container conditioned as described above. After the plates were placed in the container, one plate was removed after 5 min, and the other 30 min, followed by MTT staining. For this, MTT [(4,5-dimethyl-thiazoyl)-2,5-diphenyl-SH-tetrazolium bromide] dye solution at 0.5 mg/mL concentration in DMEM containing 2% FBS added into all wells. After 3 h of incubation at 37 °C, and 5% CO_2_, the content was replaced with DMSO to extract the formazan crystals. Subsequently, the optical density (OD) was read on a microplate reader (Tecan Sunrise, Mannedorf, Zurich, Switzerland) at 570 nm. For the calculation of the results, the mean values for the cells exposed to Coldmix^®^ vapor and untreated (control) for 5 and 30 min were recorded.

### 2.5. Statistical Analysis

In vitro data was expressed as mean ± standard error of the mean (Mean ± SEM). The statistical significance between groups was analysed by One-way ANOVA (followed by Dunnett’s post hoc test) and Paired Samples T-Test. The statistical analysis was carried out using the GraphPad Prism 7.0 and SPSS Statistics 22.0 software. The *p* < 0.05 was considered as statistically significant.

## 3. Results and Discussion

### 3.1. ACE2 Enzyme Inhibitory Activity

In the present study, the in vitro ACE2 enzyme inhibitory activity of the essential oil blend Coldmix^®^ (at 20 µg/mL concentration), and its major components 1,8-cineole as well as α-pinene (at 5 µg/mL concentration) were evaluated, respectively. α-Pinene showed a relatively higher ACE2 inhibitory activity compared to 1,8-cineole, and the Coldmix^®^ itself. While the Coldmix^®^ showed 72% ACE2 enzyme inhibitory activity, the major components 1,8-cineole and α-pinene showed 88.12%, and 80.03% enzyme inhibition, respectively, as shown in Figure 1.

The possible utilization and application of ACE2 inhibitors against COVID-19 infections were reported in previous studies [44]. As is well known, the interaction of the COVID-19 virus spike and ACE2 is necessary for virus infection; any agent that interrupts its interaction, the human monoclonal antibody based on the receptor binding domain, and the recombinant human ACE2 protein (rhuACE2) are current targets [45]. The ACE2 inhibitory activity evaluation of 1,8-cineole of the *Rosmarinus* essential oil was previously reported [28] also in correlation with the findings in this present study. In addition, in recent studies and reports [40] of our group on in vitro ACE2 enzyme inhibition of *Eucalyptus* essential oil was also relatively high, suggesting that the recent effect of Coldmix^®^ may be influenced by *Eucalyptus* oil. As Coldmix^®^ inhibited in vitro the ACE2 system, the commercial product and its major components could be effective against SARS-CoV-2. This was also supported by the in vitro antiviral effects, which is also reported in this work for the first time.

### 3.2. Antiviral Activity and Cytotoxicity of Vapor Phase

In this present work, the in vitro antiviral activity of the Coldmix^®^ was tested against the human pathogenic SARS-CoV-2 virus, where the initial results are shown in Table 1. The essential oil blend Coldmix^®^ indicated remarkable antiviral activity against the tested human pathogenic virus at the tested concentration as low as 0.1 mg/mL in the contact range of 5 to 30 min. Visually confirmed, Coldmix^®^ destroyed the virus by 79.89% in the 5th min, and 93.23% in the 30th min, as per the experimental measurements.

1,8-Cineole, a component of Coldmix^®^, is known for its antiviral potential against different human pathogen DNA and RNA viruses such as rhinoviruses, HSV-1, hepatitis A virus, herpes viruses (HSV), adenoviruses (ADV), hepatitis B virus, coxsackievirus B1 (CVB1), and enterovirus 71 (EV71) [46,47,48,49]. As a result of this in vitro study, it was observed that the product, which is rich in 1,8-cineole, is relatively effective against the coronavirus tested. It can be proposed that 1,8-cineole may contribute or is even responsible for this effect. According to previous studies, α-pinene, was tested against different human pathogenic viruses, and promising results were observed [47]. In addition, a study on mixtures of aroma substances, volatile compounds or essential oils showed that in some cases, individual volatile components showed relatively lower effects compared to their initial essential oils, respectively [50]. The medical device Coldmix^®^, is also such a unique combination of volatile components in this respect, which may have future potential in natural antiviral applications on clinical experiments.

Based to the experimental data, the tested Coldmix^®^ product was also found to be relatively safe in vitro according to the cytotoxicity data on healthy Vero cells in the vapor phase for 5 min and 30 min, using a similar setup to the antiviral assay as explained in detail. As Coldmix^®^ is generally used by inhalation, its cytotoxicity was elaborated in the vapor phase. After 5 min and 30 min of exposure, no difference was observed between the negative control group and the Coldmix^®^ test group, which suggests the relative safe use of this special essential oil blend in the vapor phase.

The COVID-19 pandemic is currently still an important and definitive health problem in the world. Essential oils are known to have a broad bioactivity spectrum such as antimicrobials and also antivirals since ancient times. There are numerous studies that reveal the potential antiviral effects of essential oils on different viruses such as polio type 1, ECHO 9, Coxsackie B1, adeno type 2, herpes simplex (HSV) type 1 and 2, and H1N1 alone or in combination [15,50,51]. Some of these viruses belong to the coronaviruses, and even though limited, there are antiviral effect studies of coronaviruses on essential oils [28,40,41,52]. The effectiveness of the proprietary commercial essential oil blend Coldmix^®^, evaluated against coronavirus, both by direct contact with the virus and by ACE2 enzyme inhibition, was demonstrated in this study for the first time. The antiviral potential of other essential oils components and volatile metabolites remain as a potential for future detailed studies.

## 4. Conclusions

The proprietary medicinal product Coldmix^®^, which has been used for more than a decade, is a special blend of essential oils containing volatile compounds which may be used both for the prevention and treatment against coronaviruses, among other microorganisms. However, further detailed bioassays as well as clinical studies are needed to confirm the efficacy against SARS-CoV-2.

## Figures and Tables

**Table 1:** The median tissue culture infective dose (TCID_50_) of Coldmix^®^ and test samples against SARS-CoV-2.

TEST	Test Material	Log TCID_50_	Mean (±)	Results	
Virus Titration	Stock Virus	7.5	-	-	
Virucidal Test (5 min)	Control D 1	6.17	6.25	R = Log K − Log T R = 0.69	72.45%
	Control D 2	6.33			
	Coldmix^®^ Test D 1	5.83	5.53		
	Coldmix^®^ Test D 2	5.50			
	Coldmix^®^ Test D 3	5.33			
Virucidal Test (30 min)	Control D 1	6.17	6.17	R = Log K − Log T R = 1.17	92.41%
	Control D 2	6.17			
	Coldmix^®^ Test D 1	4.83	5.00		
	Coldmix^®^ Test D 2	5.17			
	Coldmix^®^ Test D 3	5.00			

## Data Availability

All data are available in this article.

## List of Abbreviations

ACE2: Angiotensin Convertying Enzyme-2, ADV: Adenoviruses, BSL3: Biosafety Level 3, COPD: Chronic obstructive pulmonary disease, COVID-19: Coronavirus disease-19, CVB: coxsackievirus B1, DMEM: Dulbecco’s Minimum Essential Medium, DMSO: Dimetyhlsulfoxide, EV71: enterovirus 71, FBS: Fetal Bovine Serum, HSV: Herpes Virus, MTT: (4,5-dimethyl-thiazoyl)-2,5-diphenyl-SH-tetrazolium bromide.
